# Supplementary material for: Genome-wide identification of directed gene networks using large-scale population genomics data
Source: Nat Commun. 2018 Aug 6;9:3097. doi: 10.1038/s41467-018-05452-6 (PMC6079029; doi:10.1038/s41467-018-05452-6)
Supplement: Supplementary file 1 — Supplementary Information [file 41467_2018_5452_MOESM1_ESM.pdf]

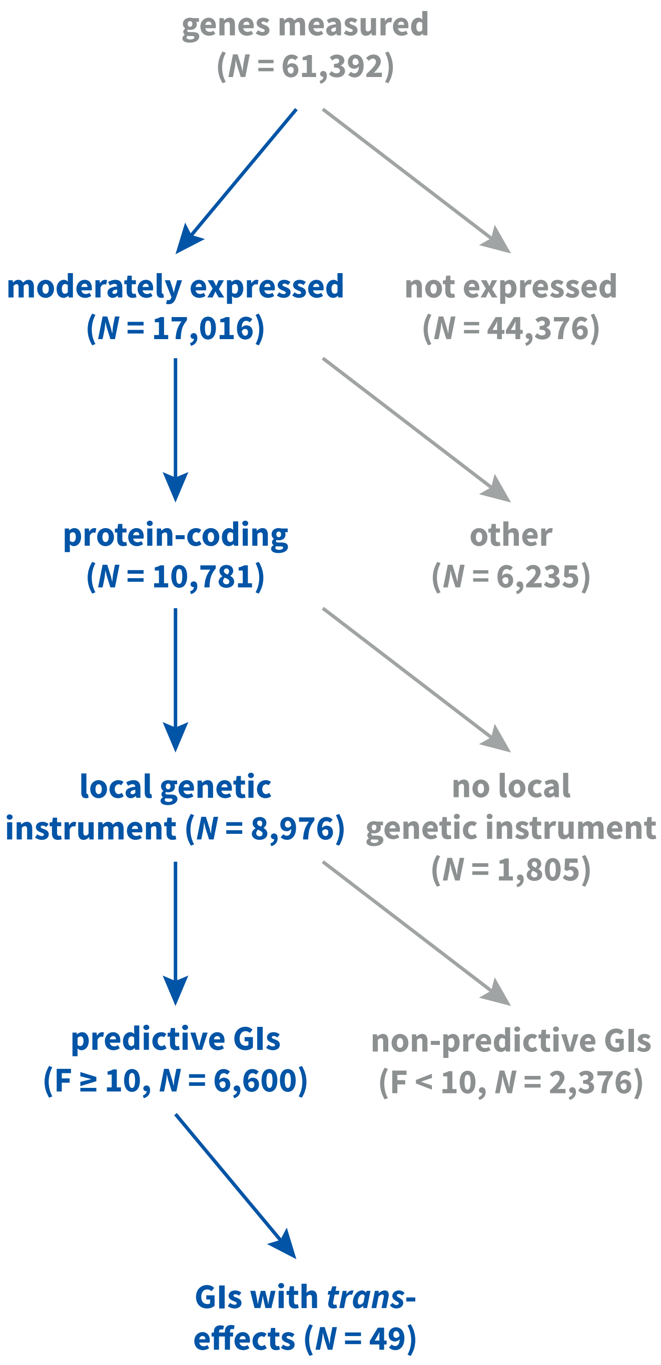

Supplementary Figure 1

Diagram showing the number of genes and genetic instruments (GIs) in each stage of the analysis.

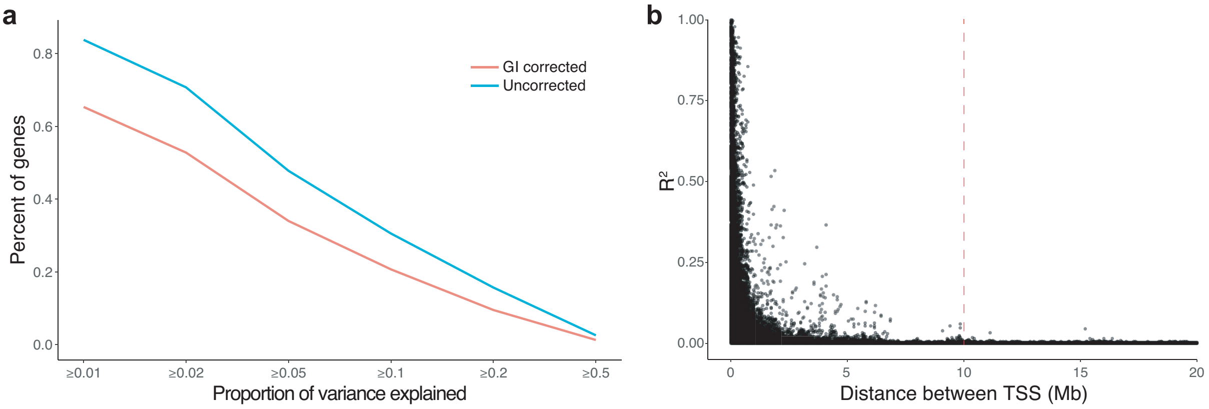

Supplementary Figure 2

Genetic instruments (GIs) account for a moderate amount of index gene expression variation explained, and are strongly correlated over small distances. a) The proportion of variance (R^2, x-axis) in index gene expression explained by the corresponding genetic instrumental variable (GI). The blue line indicates the uncorrected R^2, or the total variance explained by the GI. The red line indicates the R^2corrected for the GIs of neighbouring index genes, or the proportion of variance explained specifically by the current GI. The proportion of variance explained generally is fairly modest. b) The correlation between genetic instruments (GIs, y-axis) of different genes strongly decreases as the distance (x-axis) between the corresponding genes increases. The median R^2 between any two GIs corresponding to genes located at least 10Mb (definition of trans, indicated by red dotted line) away from each other is 1.5×10^{-4}.

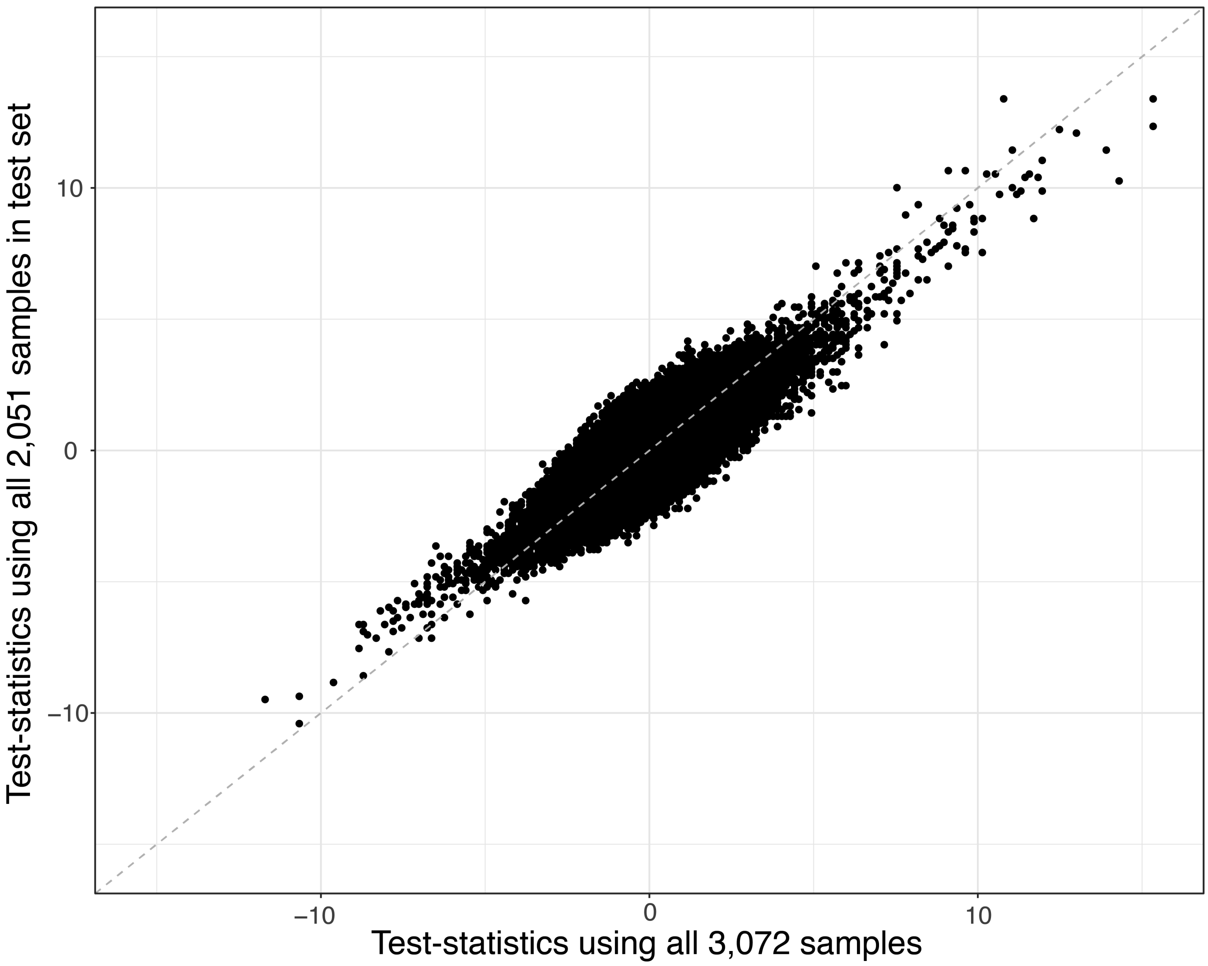

Supplementary Figure 3

Testing for an association of the 6,600 GIs with all 10,781 potential target genes when using only the 2,051 samples in the test set and when using all 3,072 samples yields similar results. The trend in test statistics suggest also using the training set does not cause bias, but does lead to an increased statistical power.

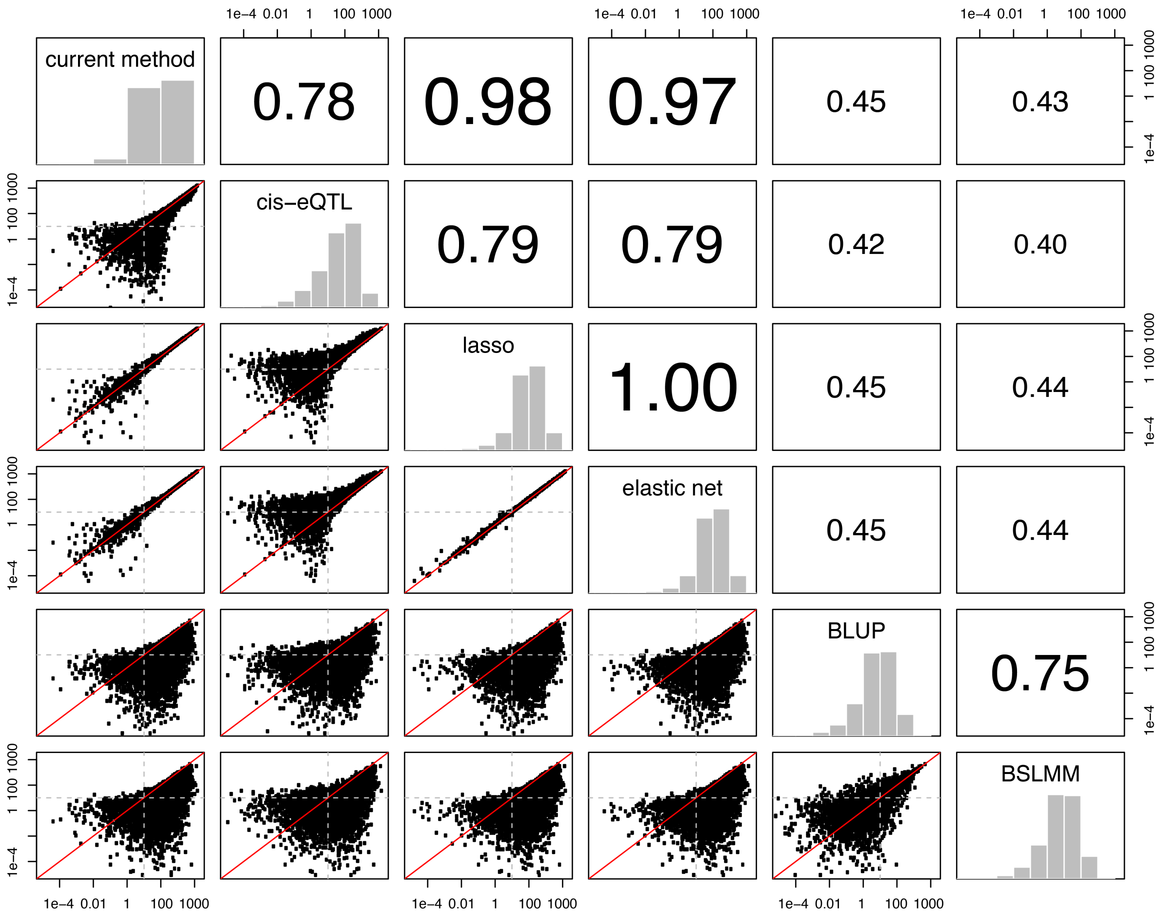

Supplementary Figure 4

F-statistics reflective of the predictive ability of the GI on the corresponding index gene. Each panel below the diagonal shows the F-statistics of two different methods plotted against each other. The panels on the diagonal show the distribution of *F*-statistics for each method. The panels above the diagonal show the Pearson correlation between the different methods. The grey horizontal and vertical lines indicate an *F*-value of 10.

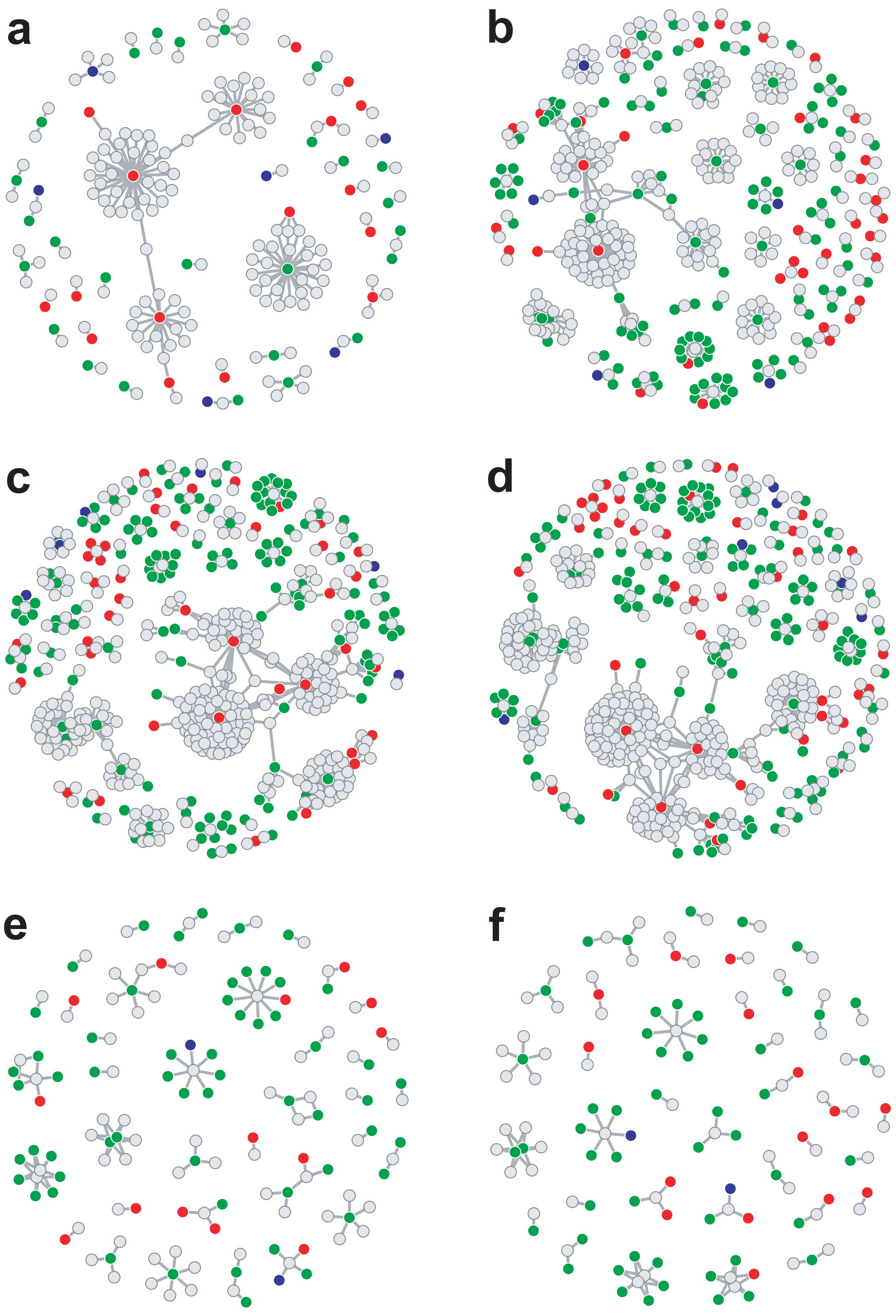

Supplementary Figure 5

Gene networks showing the directed gene-gene association between genes for the different methods. The panels show these different methods: a, our proposed method; b, top *cis*-eQTL; c, lasso; d, elastic net; e, BLUP; f, BSLMM). The lower number of *trans*-effects

identified for BLUP (e) and BSLMM (f) is likely to be reflective of their lower *F*-statistics (Supplementary Fig. 4). The other proposed methods (b-f) show a high number of target genes (grey circles) associated with the GIs of multiple index genes (colored circles). Upon closer inspection, these index genes are often neighboring, suggesting LD/pleiotropy is the cause for this observation.

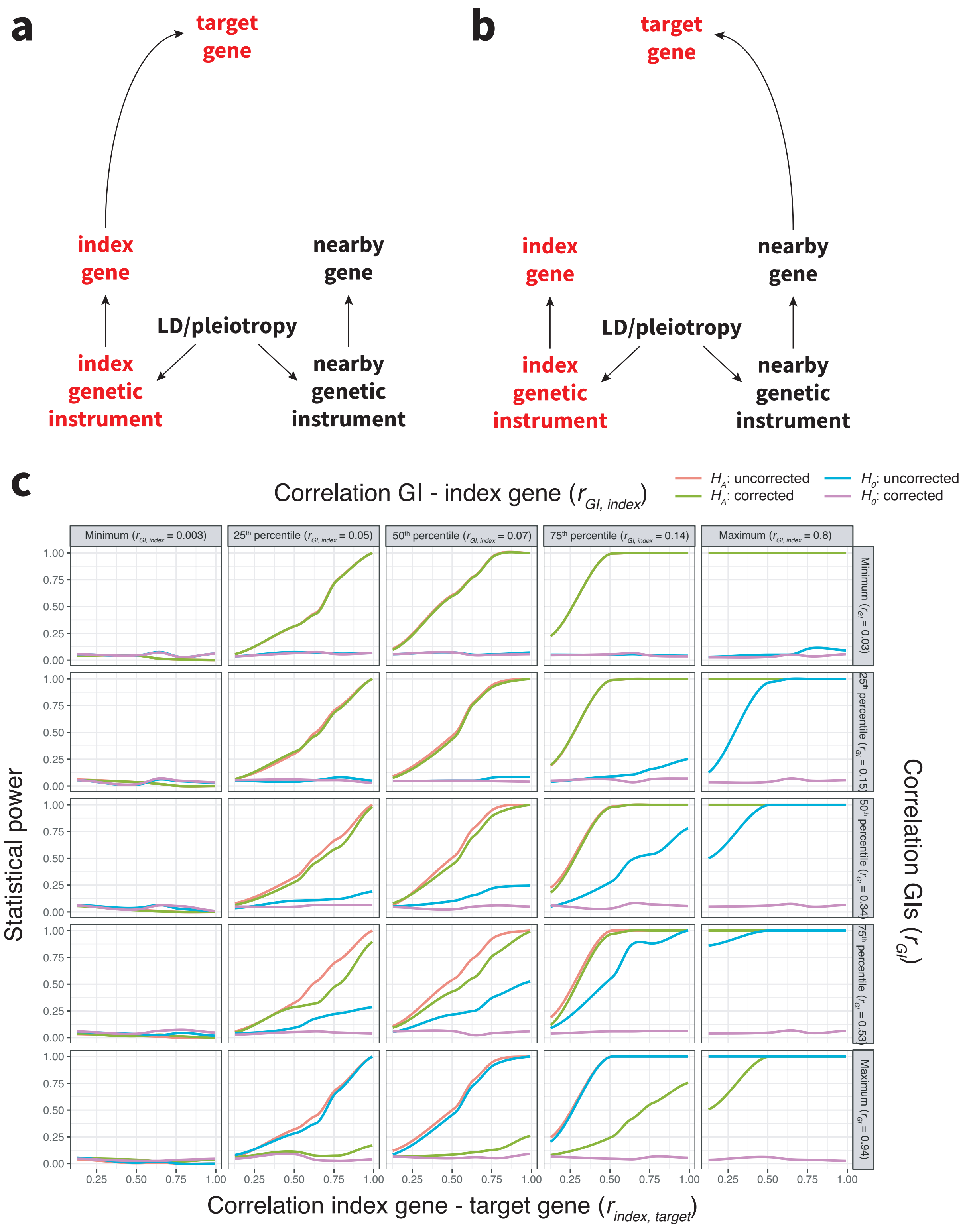

Supplementary Figure 6

Design and results of the simulation study to investigate the power of the proposed method under different circumstances. The two different scenarios represent true causality (a) and

an observed association due to LD (b). c) The statistical power for different values for the different correlations between the GIs, index gene, and target gene (r_{GI}, $r_{GI,\ index}$, $r_{GI,\ nearby}$, $r_{index,\ target}$). The red and green lines show the results under the alternative hypothesis (uncorrected for LD, and corrected for LD, respectively), i.e. when the GI of the index gene causally affects the target gene. The blue and purple show the results under the null hypothesis.

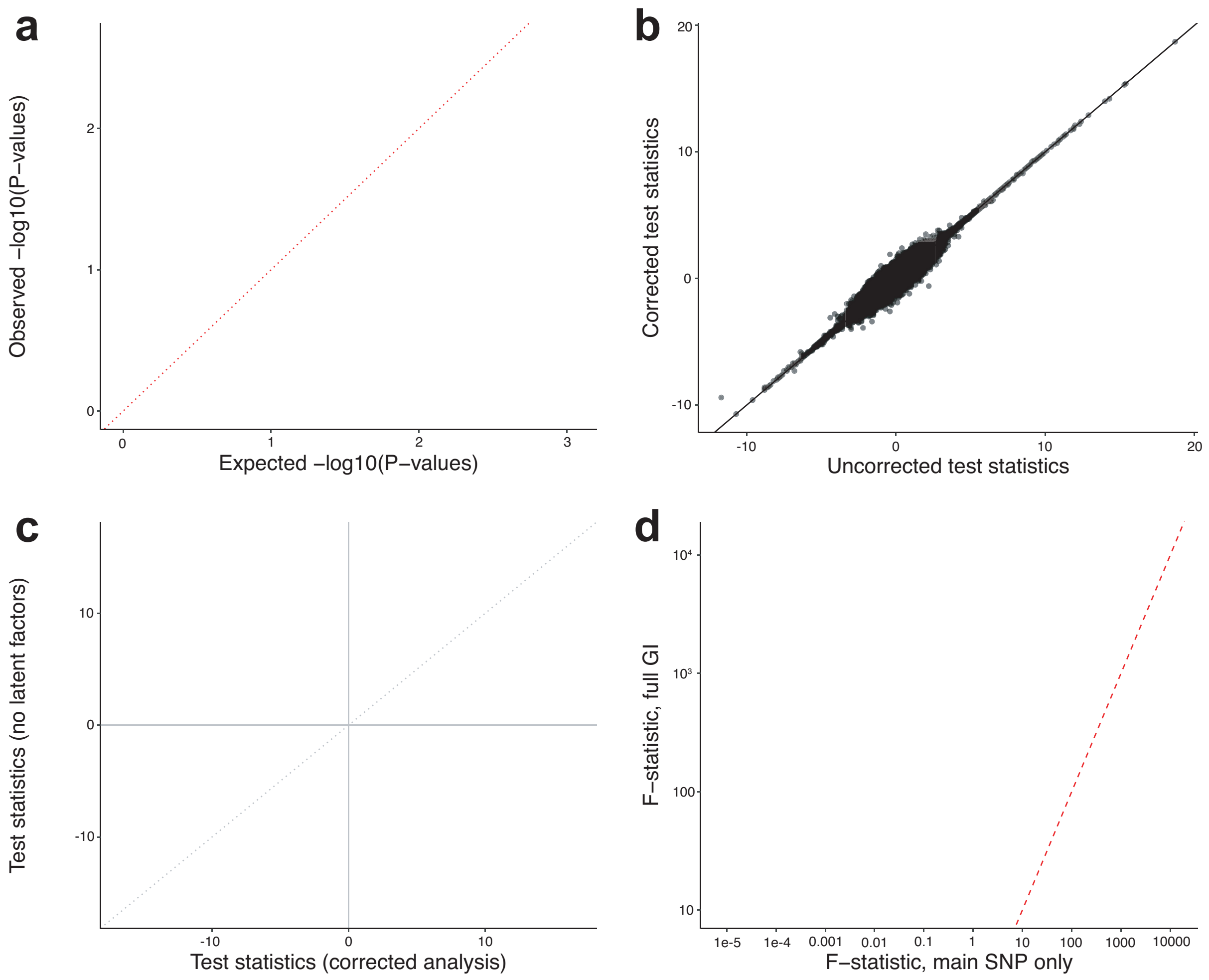

Supplementary Figure 7

Several checks indicate the stability of our analysis. a) Quantile-quantile plot of the expected $-\log_{10}$(*P*-values) (x-axis) and observed $-\log_{10}$(*P*-values) (y-axis) resulting from associating all GIs with known cell counts. The observed *P*-values follow the distribution expected under the null hypothesis, indicative of no association between the GIs and known cell counts. b) All 156 directed associations remained after further adjustment for nearby genetic variants (< 1Mb) reported to influence blood composition[15,16]. Test statistics before (x-axis) and after adjustment (y-axis) for such nearby SNPs are all along the diagonal, indicating the reported SNPs do not confound the analysis. c) Correcting for latent factors leads to slightly more significant results. Depicted are the test-statistics in the original analysis, corrected for

latent factors (x-axis), and the test-statistics without correction for these latent factors (y-axis). d) Multi-SNP GIs outperform single-SNP GIs in terms of predictive ability of index gene expression. The *F*-statistic calculated in the test set using the main, strongest associated SNP in the GIs is plotted against the *F*-statistic calculated using the full GI. Using the full GI results in 6,600 GIs predictive of the corresponding index gene (*F*-statistic > 10), whereas a single-SNP approach results in 4,910 predictive GIs.

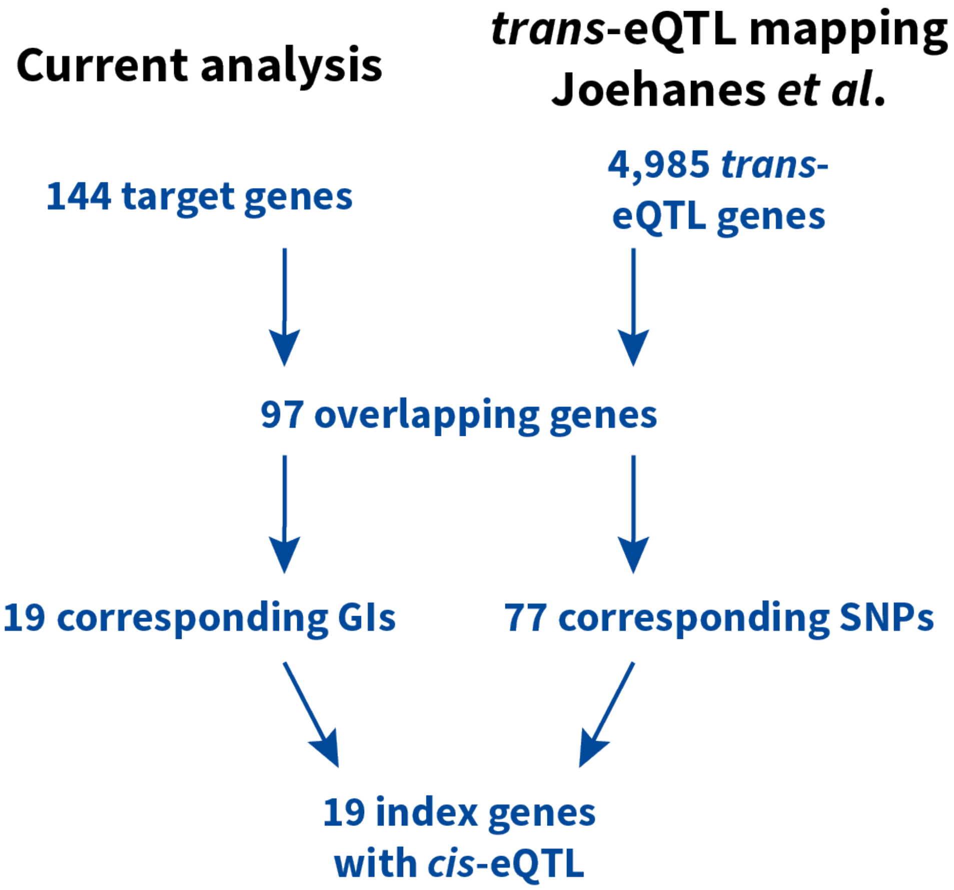

Supplementary Figure 8

Diagram comparing the identified effects in the current analysis and those identified by an earlier *trans*-eQTL mapping effort[19].

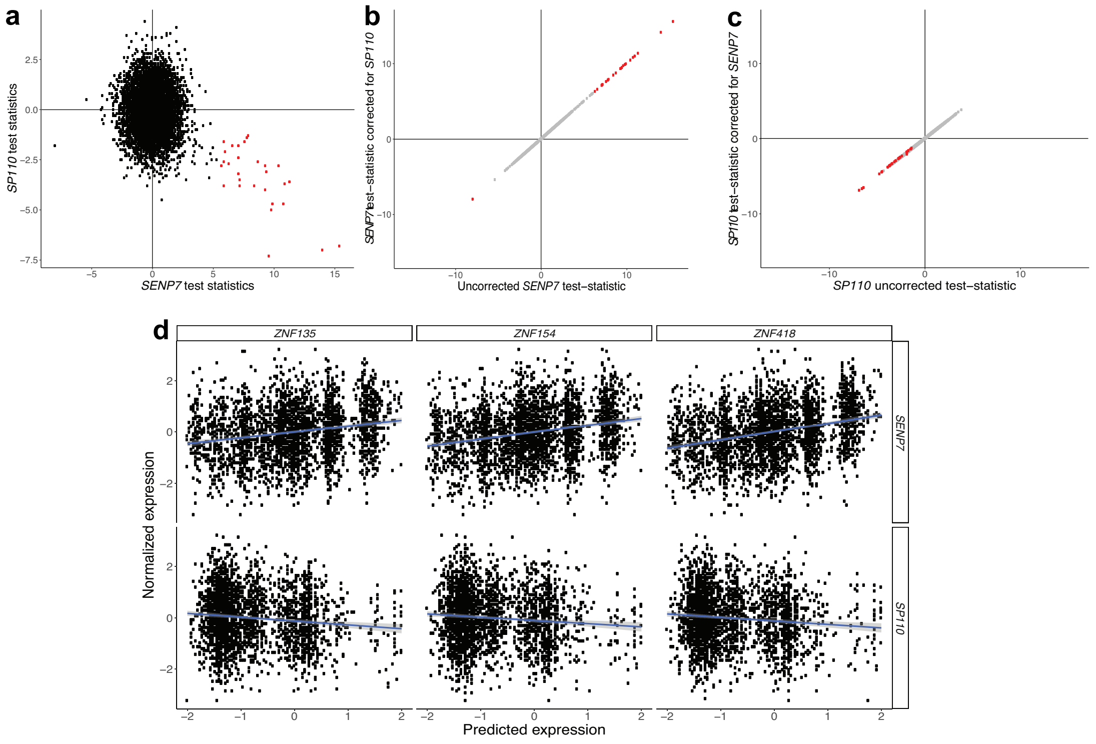

Supplementary Figure 9

SENP7 and *SP110* have shared, but opposite effects on the zinc finger protein cluster on chromosome 19. a) Test-statistics for *SENP7* and *SP110* show consistent opposite effects on the ZNF-cluster. b, c) Test-statistics of the directed effects of *SENP7* and *SP110* on target genes, correcting for each other's genetic instruments (GIs). The unchanged test-statistics indicate their effects are independent. d) Illustrations of shared, but opposite effects.
