# Supplementary material for: Genome-wide identification of directed gene networks using large-scale population genomics data
Source: Nat Commun. 2018 Aug 6;9:3097. doi: 10.1038/s41467-018-05452-6 (PMC6079029; doi:10.1038/s41467-018-05452-6)
Supplement: Supplementary file 3 — Description of Additional Supplementary Files [file 41467_2018_5452_MOESM3_ESM.pdf]

Description of Additional Supplementary Files

File Name: Supplementary Data 1
Description: The number of samples used per biobank listed after quality control.

File Name: Supplementary Data 2
Description: The predictive ability of the genetic instrument per index gene. The predictive ability is reflected by the F-statistic.

File Name: Supplementary Data 3
Description: Directed associations between genes without correcting the analysis for LD and pleiotropy.

File Name: Supplementary Data 4
Description: *Cis*-eQTLs for those index genes that also show a *trans*-eQTL effect in a recent study.

File Name: Supplementary Data 5
Description: Directly testing for a mediated effect of the index gene genetic instrument (GI) on target gene expression, where the effect is mediated by index gene expression.

File Name: Supplementary Data 6
Description: Results of the enrichment tests using GO-terms. This dataset contains both the enrichment tests performed on the directed associations found without, and with correction for LD and pleiotropy.

File Name: Supplementary Data 7
Description: Index gene-target gene pairs identified in our data that are known from different studies.

File Name: Supplementary Data 8
Description: Directed associations between genes while correcting the analysis for LD and pleiotropy.
